# The influence of implant–abutment connection on the screw loosening and microleakage

**DOI:** 10.1186/s40729-018-0121-y

**Published:** 2018-04-09

**Authors:** Katsuhiro Tsuruta, Yasunori Ayukawa, Tatsuya Matsuzaki, Masafumi Kihara, Kiyoshi Koyano

**Affiliations:** Section of Implant and Rehabilitative Dentistry, Division of Oral Rehabilitation, Faculty of Dental Science, Kyushu University, 3-1-1 Maidashi, Higashi-ku, Fukuoka, 8128582 Japan

**Keywords:** Implant–abutment connection, Microleakage, Microgap, Screw loosening, Toluidine blue, Non-axial load

## Abstract

**Background:**

There are some spaces between abutment and implant body which can be a reservoir of toxic substance, and they can penetrate into subgingival space from microgap at the implant–abutment interface. This penetration may cause periimplantitis which is known to be one of the most important factors associated with late failure. In the present study, three kinds of abutment connection system, external parallel connection (EP), internal parallel connection (IP), and internal conical connection (CC), were studied from the viewpoint of microleakage from the gap between the implant and the abutment and in connection with the loosening of abutment screw.

**Methods:**

We observed dye leakage from abutment screw hole to outside through microgap under the excessive compressive and tensile load and evaluated the anti-leakage characteristics of these connection systems.

**Results:**

During the experiment, one abutment screw for EP and two screws for IP, out of seven samples in each group, were fractured. After the 2000 cycles of compressive tensile loadings, removal torque value (RTV) of abutment screw represented no statistical differences among three groups. Standard deviation was largest in the RTV of EP and smallest in that of CC. The results of microleakage of toluidine blue from implant–abutment connection indicated that microleakage generally increased as loading procedure progressed.

The amount of microleakage was almost plateau at 2000 cycles in CC, but still increasing in other two groups. The value of microleakage greatly scattered in EP, but the deviation of that in CC is significantly smaller. At 500 cycles of loading, there were no significant differences in the amount of microleakage among the groups, but at 1000, 1500, and 2000 cycles of loading, the amount of microleakage in CC was significantly smaller than that in IP. Throughout the experiment, the amount of microleakage in EP was largest, but no statistical difference was indicated due to the high standard deviation.

**Conclusions:**

Within the limitation of the present study, CC was stable even after the loading in the RTV of abutment screw and it prevented microleakage from the microgap between the implant body and the abutment, among the three tested connections.

## Background

Although promising outcome of implant therapy has been reported, periimplantitis which is known to be one of the most important factors associated with late failure [1] is a serious complication and expected to overcome to obtain successful outcome. It is known that there are some spaces between abutment and implant body which can be a reservoir of microorganisms and other toxic substance [2], and they can penetrate into subgingival space from microgap located at the implant–abutment interface. This is believed to impact to periimplant inflammation [3]. Thus, many kinds of implant–abutment connection have been proposed to minimize the microgap [4].

Implant–abutment connection can be divided into three types, external parallel connection (EP), internal parallel connection (IP), and conical connection which has the friction between the implant and the abutment (CC). Nobel Biocare has implant systems with these three kinds of implant–abutment connections, and these connection systems are popular even in other manufacturers and they also employ one or some of these connection systems. These systems have a lot of pros and cons and should be selected depending on the dentists’ demand.

In the present study, we would like to consider these three kinds of connection system from the point of view of microleakage from the gap between the implant and the abutment and in connection with the loosening of abutment screw. We herewith performed the investigation of dye leakage from abutment screw hole to outside through microgap under the excessive cyclic load applied to cantilever superstructure. Cantilever model could simulate non-axial offset loading applied to implant–abutment complex, and toluidine blue solution was used to measure the extravasation from the gap at the implant–abutment interface.

## Methods

**Fig. 1:**
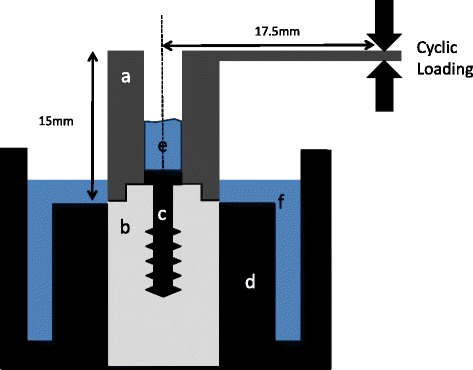
A scheme of experimental assembly. a: Abutment and superstructure; b: implant body; c: abutment screw; d: steel mold; e: toluidine blue solution; and f: water

Three kinds of Nobel Biocare implants were employed in the present study, namely, Nobel SpeedyGroovy WP 5.0 × 15 mm (EP), Nobel Replace WP 5.0 × 15 mm (IP), and Nobel Parallel CC RP 5.0 × 15 mm (CC) (*n* = 7 each) (Nobel Biocare, Kloten, Switzerland). Implant–abutment connections are external, internal parallel, and internal and conical connection, respectively. Implant was embedded into steel mold and fixed with epoxy resin (Araldite®, Nichiban, Tokyo, Japan). A superstructure with cantilever (height 15 mm; length 20 mm from the center of access hole) was fabricated using Au–Pt alloy (Degudent LTG, Degudent, Hanau-Wolfgang, Germany) and fixed with abutment screw with fastening torque recommended by the manufacturer (35 Ncm), using torque wrench (Nobel Biocare). Five hundred microliter of water was poured into steel mold, the surface of water located, to small extent, superior to microgap. Then, 50 μl of 0.05% toluidine blue solution was poured at the access hole (Fig. 1).

This assembly was set in a universal test machine (Autograph AS-1S, Shimadzu, Kyoto, Japan), and load was applied to the cantilever, at 17.5 mm distance from the center of access hole.

As one cycle, one compressive and tensile loads (10 N each) was applied per 1 s and 2000 cycles of loading was done. Before starting load application and every 100 cycles, 100 μl solutions were collected from pool and absorbance at 627 nm was measured using a spectrophotometer (Biospec-mini, Shimadzu). Every 500 cycles, the amount of microleakage was statistically compared using Student *t* test with Bonferroni correction for multiple comparisons. After the completion of 2000-cycle loading, removal torque value (RTV) of abutment screw was measured.

## Results

During the experiment, one (EP) and two (IP) abutment screws out of seven samples were fractured. In this case, abutment screw was changed to new one and experimental procedure was re-run.

### RTV of abutment screw after 2000-cycle loading

**Fig. 2:**
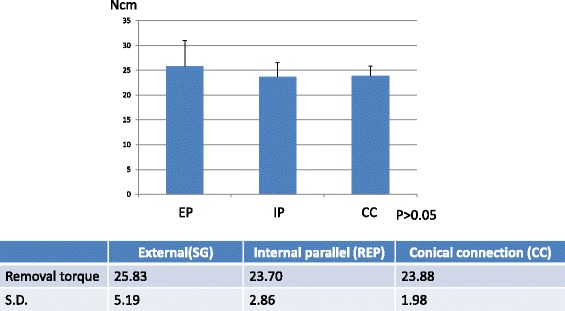
Removal torque of abutment screw after 2000-cycle loading

After the 2000 cycles of compressive tensile loadings, RTV of abutment screw was measured. There were no statistical differences in the RTV among three groups. Standard deviation was largest in the RTV of EP and smallest in that of CC (Fig. 2).

### Microleakage of toluidine blue from implant–abutment connection

**Fig. 3:**
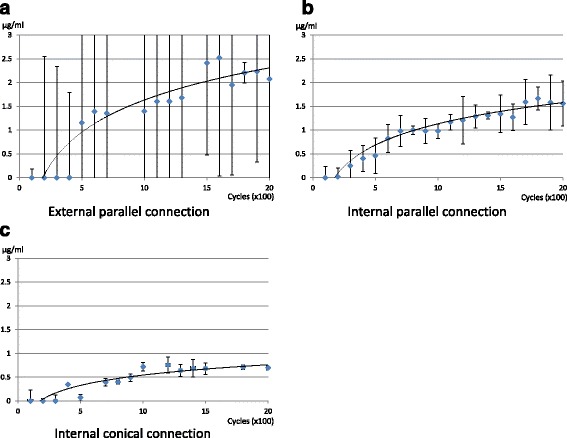
**a** Microleakage from external parallel connection implant–abutment connection. Vertical bars indicate standard deviation. **b** Microleakage from internal parallel connection implant–abutment connection. Vertical bars indicate standard deviation. **c** Microleakage from internal conical connection implant–abutment connection. Vertical bars indicate standard deviation

All groups indicated that microleakage generally increased as loading procedure progressed (Fig. 3) and logarithmic trendline could be drawn, with *R*^2^ values of 0.854 in EP, 0.924 in IP, and 0.847 in CC. The amount of microleakage was almost plateau at 2000 cycles in CC group (Fig. 3c), but still increasing in other two groups (Fig. 3a, b). The value of microleakage greatly scattered in EP groups (Fig. 3a), but the deviation of that in CC group is significantly smaller (Fig. 3c).

**Fig. 4:**
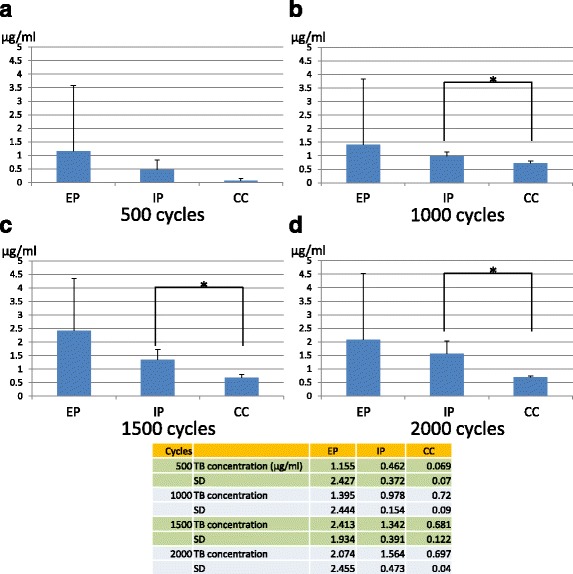
**a** Microleakage of toluidine blue from implant–abutment connection at 500 cycles of loading. No statistical difference is indicated (*P* > 0.05). **b** Microleakage of toluidine blue from implant–abutment connection at 1000 cycles of loading. **c** Microleakage of toluidine blue from implant–abutment connection at 1500 cycles of loading. **d** Microleakage of toluidine blue from implant–abutment connection at 2000 cycles of loading. EP external parallel connection, IP internal parallel connection, CC internal conical connection. Asterisk indicates statistically significant difference (*P* < 0.05)

At 500 cycles of loading, there were no significant differences in the amount of microleakage among the groups (Fig. 4a), But at 1000, 1500, and 2000 cycles of loading, the amount of microleakage in CC group was significantly smaller than that in IP group (Fig. 4b–d). There were no statistical differences between EP and other groups in every measurement (Fig. 4).

## Discussion

In the present study, cyclic tensile and compressive loading were applied to cantilever superstructure. In the clinical situation, both compressive and tensile force was exerted to implant-supported prosthesis, but no previous study has discussed the microleakage using model study under this situation. In case of conical connection, compressive force may have promoted a higher penetration of the abutments into implant body, which may eliminate microgap [5]. But in the clinical situation, tensile force may also be applied to abutment–implant interface as indicated above; the model employed in the present study may be pertinent.

Some previous model studies which measured the extent of microleakage from implant–abutment interface employed microorganisms [6–9]. These studies focused on bacteria itself using visible solution cloudiness test [6], scanning electron microscopy [7], bacteria viability test [8], or checkerboard DNA–DNA hybridization method [9] and did not mentioned about the bacterial toxin. Toluidine blue employed in the present study can easily be measured using absorptiometry and it was reported to be similar to bacterial toxins in its molecular size [5]. In addition, trend in microleakage was reportedly similar between bacterial leakage model and dye leakage model [10].

In the present study, removal toque of abutment screw after the cyclic loading showed no statistically significant difference among the groups. Generally, conical abutment is believed to be better in fit and stability than non-conical connection [11]. One possibility of this discrepancy may be due to the deformation of abutment screw. Actually, implant–abutment connection after the removal of abutment screw was still tight in CC group, but they were easily divided in other two groups. This may indicate that implant–abutment connection in CC group was almost sound and intact after the loading. In contrast, in EP and IP groups, abutment screw may be deformed which lead to the increase of RTV of abutment screw. The fracture of abutment screws in EP and IP groups may support this speculation. In addition, axial force is strongly affected by the interfacial friction coefficient [12]. In the present study, interfacial friction was supposed to be largest in CC group because the contact area between implant body and abutment was smallest in EP group and both EP and IP groups had parallel walls at the interface with gaps and voids [13, 14]. This may be one reason for the larger standard deviation of RTV in both EP and IP groups.

In the present study, chronological increase of the amount of microleakage was observed in all three groups. This is in consistent with previous studies [5, 13, 15, 16]. Sigmoid curves of microleakage in all groups meant that the amount of leakage was large at the early stage of loading. This was in agreement with previous reports. Harder et al. reported that bacterial toxin leak occurred within 5 min of incubation using in vitro experimental model study, even without application of loading [15, 16].

The comparison of microleakage among the groups at every 500-cycle load indicated that there were no significant differences among the groups at 500-cycle loading but were statistically significant differences in those between IP and CC groups. The reason for microleakage in EP group having no significant differences indicated between EP and IP or CC groups may be due to the largeness of standard deviation in the value of EP group.

The limitation of the present study was the number of samples. We believed the sample number (seven in each group) was sufficient to obtain the trends of microleakage, but it may be better to investigate using large number of samples to analyze the nature of microleakage at the implant–abutment interface in detail. Another limitation was that the fastening torque applied to screw was not necessarily accurate. To mimic clinical situation, we used dedicated beam-type toque wrench delivered from manufacturer. According to the previous studies, the wrench of Nobel Biocare only reportedly demonstrated the target torque value falling within the 95% confidence interval for the true population mean among four kinds of wrenches [17]. In addition, significantly lower deviations of torque values for beam-type wrenches were reported than for coil and toggle-style wrenches [18]. But toque wrench still has an inaccuracy because the scale printed on the wrench is not fine and it seems to be inappropriate to apply precise torque value. In the present study, we used new torque wrench, but in the clinical settings, variability of toque value may expand because torque wrench is repeatedly used, with sterilization procedure, which decrease the accuracy [19].

## Conclusions

Within the limitation of the present study, after the offset cyclic tensile and compressive loading, the amount of microleakage from implant–abutment interface was smaller in conical connection than in internal parallel connection.
